# Human T-cell Leukemia Virus Type 1 and *Strongyloides stercoralis*: Partners in Pathogenesis

**DOI:** 10.3390/pathogens9110904

**Published:** 2020-10-29

**Authors:** Adam Dykie, Tharaka Wijesinghe, Arnold B. Rabson, Kiran Madugula, Christian Farinas, Sydney Wilson, David Abraham, Pooja Jain

**Affiliations:** 1 Department of Microbiology and Immunology, Drexel University College of Medicine, Philadelphia, PA 19102, USA; agd48@drexel.edu (A.D.); trw54@drexel.edu (T.W.); km3349@drexel.edu (K.M.); caf349@drexel.edu (C.F.); ssw56@drexel.edu (S.W.); 2 Child Health Institute of New Jersey and Departments of Pharmacology, Pediatrics and Pathology and Laboratory Medicine, Rutgers Robert Wood Johnson Medical School, New Brunswick, NJ 08901, USA; rabsonab@rwjms.rutgers.edu; 3 Department of Microbiology and Immunology, Sidney Kimmel Medical College at Thomas Jefferson University, Philadelphia, PA 19107, USA; David.Abraham@jefferson.edu

**Keywords:** HTLV-1, ATLL, HAM/TSP, strongyloidiasis, co-infection

## Abstract

Infection with human T-cell leukemia/lymphoma virus type 1 (HTLV-1) has been associated with various clinical syndromes including co-infection with *Strongyloides stercoralis*, which is an intestinal parasitic nematode and the leading cause of strongyloidiasis in humans. Interestingly, HTLV-1 endemic areas coincide with regions citing high prevalence of *S. stercoralis* infection, making these communities optimal for elucidating the pathogenesis of co-infection and its clinical significance. HTLV-1 co-infection with *S. stercoralis* has been observed for decades in a number of published patient cases and case series; however, the implications of this co-infection remain elusive. Thus far, data suggest that *S. stercoralis* increases proviral load in patients co-infected with HTLV-1 compared to HTLV-1 infection alone. Furthermore, co-infection with HTLV-1 has been associated with shifting the immune response from Th2 to Th1, affecting the ability of the immune system to address the helminth infection. Thus, despite this well-known association, further research is required to fully elucidate the impact of each pathogen on disease manifestations in co-infected patients. This review provides an analytical view of studies that have evaluated the variation within HTLV-1 patients in susceptibility to *S. stercoralis* infection, as well as the effects of strongyloidiasis on HTLV-1 pathogenesis. Further, it provides a compilation of available clinical reports on the epidemiology and pathology of HTLV-1 with parasitic co-infection as well as data from mechanistic studies suggesting possible immunopathogenic mechanisms. Furthermore, specific areas of potential future research have been highlighted to facilitate advancing understanding of the complex interactions between these two pathogens.

## 1. Introduction

A well-characterized disease manifestation associated with human T-cell leukemia/lymphoma virus type 1 (HTLV-1) is the co-infection with *Strongyloides stercoralis*. Although the mechanism of the co-infection is not fully understood, it is possible that the microorganisms interact in a complicated manner to influence the course of the combined disease. The epidemiology of HTLV-1 infection is intimately intertwined with its biology. Although worldwide prevalence of HTLV-1 is not at the levels seen for the epidemic of human immunodeficiency virus type 1 (HIV-1), HTLV-1 infection is nonetheless a significant problem affecting millions of individuals across the globe. While the exact numbers of people infected with HTLV-1 remain uncertain, estimates vary from a minimum of 5–10 million, based on data from epidemiologic studies covering areas with approximately 1.5 billion people [1], to as many as 20 million infected worldwide [2,3].

The prevalence of infection is heterogeneous across different populations, often achieving high levels (as high as 5–40%) in small population niches, even within an individual country (reviewed in [1,3]). Compared to HIV-1, HTLV-1 is relatively poorly infectious, relying on direct cell-to-cell transmission to spread the virus [4]. Given this feature, infected lymphocytes must be directly transmitted for efficient infection. Therefore, the most common modes are breast milk transmission of infected cells from mother to child, sexual transmission primarily male to female, and transmission from blood and blood products such as intravenous drug abuse [5]. Breast milk transmission is preventable in societies in which formula feeding is a practical and healthy alternative, such as Japan, which has seen a marked decrease in perinatal transmission in endemic areas [6]. These modes of transmission have also likely impacted the geographic distribution of this virus. Geographical clustering of HTLV-1 infection is well-known, dating back to its initial discovery. In fact, the clustering of adult T-cell leukemia/lymphoma (ATLL) cases in southwestern Japan was a sentinel observation suggesting the existence of a potential new human oncogenic virus [7]. In 1980, Poiesz et al. identified HTLV-1 from a U.S. patient with a similar CD4+ T-cell lymphoma [8]. Similarly, Yoshida and colleagues isolated HTLV-1 from ATLL patients, clearly identifying the virus as the causative agent of this malignancy [9]. Subsequently, the high levels of HTLV-1 both in Japan and in the Caribbean islands led to its identification as the cause of the demyelinating disease referred to as HTLV-associated myelopathy (HAM, originally described in Japan) or tropical spastic paraparesis (TSP, identified in patients of Caribbean origin) [10,11]. Other areas of endemic infection include regions of Sub-Saharan Africa, much of South America, and focal infection in the Middle East and in Australia and Melanesia.

**Figure 1:**
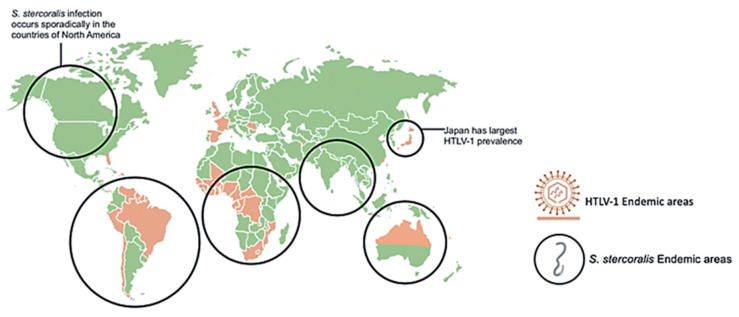
Geographical distribution of human T-cell leukemia/lymphoma virus type 1 (HTLV-1) and *Strongyloides stercoralis* with overlapping areas of prevalence. Areas highly prevalent with HLTV-1 infection include Japan, Northern Australia, Africa, and South America. Areas highly prevalent with *S. stercoralis* infection include tropical and sub-tropical areas across the globe including sporadic infection in North America. *S. stercoralis* image retrieved from https://www.cdc.gov/dpdx/strongyloidiasis/index.html.

Since infected individuals are concentrated in discrete geographical locations, and the testing for HTLV-1 across much of the world is often unreliable, there is significant uncertainty in the global extent of HTLV-1 infection. Furthermore, many of the serological prevalence studies have used specific subpopulations, including blood donors or pregnant women seen at specific health care facilities. Data from such groups may not be representative of the general population as a whole. HTLV-1 co-infections with other pathogens, such as nematode *S. stercoralis*, have been appreciated for over three decades [12,13,14,15]. Studies of areas of high prevalence of both infections including case reports of co-infected patients, as represented in Figure 1, provided insights on the interactions between these two pathogens [16,17,18,19,20,21,22,23,24,25,26,27,28,29,30,31,32,33,34]. The geographic overlap of many areas of endemic infection for each pathogen have raised the question of whether HTLV-1 infection increases the incidence of *S. stercoralis* infection, or whether conversely, infection with this nematode might increase the incidence of HTLV-1 infection. Alternatively, it is possible that neither infection specifically alters the incidence of the other, but that co-infection may change the clinical picture of either infection. Overall, data support the later conclusion, including the possibilities that *S. stercoralis* infection might accelerate the onset of HTLV-1 disease, and that HTLV-1 infection is associated with a higher likelihood of higher parasite burden, more clinically apparent infection, and more severe and life-threatening disease [12,13,35].

This paper explores the pathophysiology of the co-infection between HTLV-1 and *S. stercoralis*. Studies are reviewed to assess the potential role *S. stercoralis* has in modulating HTLV-1, and subsequently, how HTLV-1 affects the immunological response to strongyloidiasis. A prospective analysis of the clinical significance and pathogenic mechanisms of this co-infection may be studied through populations with a high infectious burden of HTLV-1 and *S. stercoralis*. Analysis of these studies will help elucidate a potential mechanism for the onset of the co-infection. Final perspectives assess the implications of the combined disease and identify areas of research and model development needed to better understand the co-infection. This may further determine whether HTLV-1 and *S. stercoralis* are indeed partners in disease pathogenesis.

*Strongyloides* is an intestinal nematode that may cause parasitic infection of humans. Estimates of global prevalence range between 100–370 million people currently infected, mostly in tropical and subtropical regions of the world [36,37,38]. The wide discrepancy in these estimates is explained in part by the difficulty in diagnosing these infections, the chronic nature of the disease, and the absence of pathognomonic signs and symptoms. As a soil transmitted helminth (STH), the infection is initiated by the invasion of the skin by the infective third stage larvae (L3). The parasites migrate extensively through the body, pass through the lungs, and develop into parthenogenic parasitic female worms in the small intestine. The adult worms release eggs, which eventually form first stage larvae (L1) in the feces. The L1 have three possible developmental pathways. The first is direct development on the ground through the transition of L1 into the infectious form L3, which can then invade the next host. In the second pathway, the L1 released on the ground develop into free living male and female adult worms that mate and produce offspring that eventually develop into infective L3. This developmental pathway ensures an abundance of larvae in the environment and thus enhances transmission of the infection to the next host. In the third pathway of development, parasites develop from L1 into autoinfective third stage larvae (L3a) without leaving the host, with the L3a reinfecting the same host. The transition from L1 to L3a and ultimately parthenogenetic female worms occurs at a slow rate, replenishing worms in the intestine as they die off. The result is not an increase in the number of adult worms in the intestine, but rather extremely long-term infections in the host, caused by the replenishing of the adult forms in the intestine for decades after the initial infection. Chronic infection with *S. stercoralis* typically presents as urticaria, diarrhea and abdominal pain, although these associations are not universal [39,40,41]. Hyperinfection may result if the infected individual is treated with steroids or is infected with HTLV-1 due to a disruption in the homeostatic relationship between the parasite and the host. Instead of parasites leaving the host in the feces as L1, they develop into L3a, exit the intestine in large numbers and disseminate to virtually every organ system with accompanying intestinal bacteria. Hyperinfection due to *S. stercoralis* results in multiple potential clinical manifestations with life-threatening consequences resulting in mortality rates of up to 85% to 100% [39,41,42,43]. Despite having separate clinical manifestations, the two pathogens appear to be closely associated, given the prevalence of the co-infection and the apparent clinical association of HTLV-1 and *S. stercoralis* hyperinfection. Characterizing the co-infection will require exploring the epidemiology of HTLV and *S. stercoralis* individually and considering each pathogen’s biology.

## 2. The Clinical Spectrum of HTLV-1 Infection

The clinical presentations of HTLV-1 infection are broad and varied and have been extensively and elegantly reviewed, as have the key principles developed in the extensive literature on HTLV-1 pathogenesis, so only a limited number of salient features will be addressed here [44,45,46,47]. While less than 10% of infected individuals develop known HTLV-1-associated diseases, even those who do not exhibit overt HTLV-1 disease do exhibit shortened lifespans. While the mechanisms responsible for this increased overall mortality are not clear, HTLV-1 infection leads to a chronic inflammatory state with a significant on-going immune response to the virus. These increased death rates are potentially associated with chronic inflammation, resulting in a well-studied increase in the morbidity of cardiovascular disease and non-lymphoid malignancies [44,45].

The most dramatic presentation of HTLV-1 infection is ATLL, an aggressive malignancy of CD4+ T-cells. ATLL occurs in approximately 3–5% of HTLV-1 infected patients, often through breast milk transmission [46]. ATLL can present as a fulminant CD4+ T-cell leukemia, an aggressive lymphoma, or as a more indolent smoldering or chronic ATLL [47,48]. Prognosis of aggressive disease remains dismal, since the average survival times of the acute leukemias and lymphomas remain in the range of 8–10 months [49]. Due to the severity of this disease, therapy for HTLV-1 malignant disease is a rapidly evolving field [50,51]. Classical chemotherapy approaches have relatively little success, and the only potential cures to date follow allogeneic hematopoietic stem cell transplantation, resulting in reconstitution of the lymphoid population [52]. Over the past two decades, protocols using the anti-viral drugs, interferon-α and azidothymidine, have shown evidence of efficacy in inducing longer remissions in acute and chronic subtypes of ATLL, including up to 100% 5-year survival in the chronic subtypes [53]. Anti-proliferative and pro-apoptotic effects of the combination of these two agents appear to be the major contributors to their real, though limited therapeutic effects [54,55].

Another promising approach for ATLL therapy has been the use of antibodies directed against receptors present in high levels on the surface of HTLV-1 malignant T-cells. Early studies targeted the interleukin-2 (IL-2) receptor alpha subunit, anti-CD25, with some success, particularly using antibodies coupled to cytotoxic agents such as cytotoxic toxin subunits [56,57]. More recently, the C-C chemokine receptor type 4 (CCR4) has been shown to be a useful target for antibody directed therapy against ATLL. CCR4 is overexpressed and/or mutated in a significant proportion of ATLL, resulting in constitutive activation of downstream signaling [58,59]. CCR4 is also expressed on other, non-malignant T-cells infected by HTLV-1 [60]. A variety of studies assessing the effects of histone deacetylase inhibitors, arsenate derivatives, the immunomodulator lenalidomide, and other antibodies and small molecules are also in progress [47,50,51].

In addition to malignant disease, HTLTV-1 infection can result in several different inflammatory diseases that may resemble autoimmune disorders. The most prevalent of these inflammatory disorders is HAM/TSP, a demyelinating disorder involving primarily the spinal cord, found in up to 2–4% of HTLV-1-infected patients [60,61]. HAM/TSP occurrence is generally associated with infection in adulthood through sexual transmission or blood transfusion. It is important to note that patients with juvenile HAM/TSP have been seen [62,63] and that ATLL is a major cause of death in HAM/TSP patients [64], suggesting that the transmission and pathogenesis of these two classes of HTLV-1 disease may not be entirely unrelated. Prior to onset of symptoms, clinical latency is typically anywhere from a couple of years to decades following infection (reviewed in [65,66]). Subsequently, HAM/TSP initially presents in the lower extremities with chronic progression of spasticity and weakness (paraparesis), contributing to increasing gait disturbance and loss of mobility. Over time, patients can experience bladder dysfunction and increasing sensory deficits. Histologically, the disease is characterized by lymphoid inflammatory infiltrates throughout the central nervous system with accompanying demyelination, axon loss, and formation of gliotic scars.

The major treatment of HAM/TSP is corticosteroids, which despite years of use, has only recently demonstrated responses in enough patients to be deemed clinically effective [67]. A little over half of patients have short-term responses, while two-thirds have longer term stable or improved disease. Nonetheless, significant numbers of patients have little or no long-term improvement or become refractory to steroid therapy. One therapy of note, mogamulizumab (anti-CCR4 antibody), has been recently reported to reduce the symptoms of spasticity and motor deficits, the presence of cerebrospinal fluid inflammatory markers and the levels of HTLV-1 proviral load. This antibody may work by targeting both CD4+CCR4+ and CD8+CCR4+ T-cell populations, both of which were shown to be reservoirs containing HTLV-1 proviruses in HAM/TSP patients [60], suggesting a promising new therapeutic approach for HAM/TSP [68] as well as ATLL.

HTLV-1 can also be associated with a generalized cytokine dysregulation and immunodeficiency, resulting in accentuation of other infectious processes in some HTLV-1 infected individuals. One notable example is HTLV-1-associated infective dermatitis, an eczematous rash, predominantly found in HTLV-1 infected children. This disease manifestation is characterized by pronounced lymphoid infiltration of the skin and associated with overgrowth of common pathogenic skin bacteria, particularly *Staphylococcus aureus* and β-hemolytic streptococci (reviewed in [69]). While this infective dermatitis is generally quite responsive to antibiotic treatment, it may be an early indicator of an underlying HTLV-1 infection and has been suspected to be a precursor to other HTLV-1-associated diseases [70]. The best characterized of these interactions between HTLV-1 and other infectious processes is the well-documented association between HTLV-1 and *S. stercoralis.*

## 3. Case Reports of HTLV-1 and *S. stercoralis* Co-Infection

Concurrent with initial serologic studies demonstrating co-infection [14,15], clinicians began to describe case reports and small series of patients exhibiting manifestations of both HTLV-1 and *Strongyloides* co-infections. As expected, patients were identified primarily in areas where HTLV-1 is endemic, and reports of co-infections span a wide patient demographic. Risk factors for strongyloidiasis, in addition to HTLV-1 include corticosteroid use and alcoholism, which often contribute to disease prognosis. Although general helminth infection is associated with HTLV-1 infection [71,72], *S. stercoralis* in particular was identified in the majority of these cases. Thus, case reports and small series have provided a window into the range of manifestations of co-infections as well as of the populations at risk. These cases highlight the broadening geographic distribution of the co-infection, the risk factors present in these patients, the unique disease manifestations, and the changing approach to treatment.

As the occurrence of HTLV-1 is increasing in areas that were once not considered endemic areas, as a result of immigration and population migrations, co-infection with helminths is occurring in these areas as well. For example, an older patient, who had immigrated to Canada from the Virgin Islands thirty years prior, exhibited co-infection [29]. HTLV-1 and *S. stercoralis* co-infection was also observed in immigrants to the UK and to the Netherlands, from Central and South America, respectively. North American immigrants from Trinidad and Jamaica have exhibited co-infection decades following exposure [30]. In the latter study, two patients exhibited unique disseminated *S. stercoralis* throughout the central nervous system, which was presumably enhanced by the HTLV-1 infection and corticosteroid use [30].

Strongyloidiasis often manifests in individuals treated with corticosteroids, although often underlying HTLV-1 infection is also present as a risk factor. Corticosteroids has been historically associated more with helminthic infection, and the risk factors are often conflated. For example, corticosteroids are a main stay of therapy for HAM/TSP, thus likely providing an additional risk factor for aggressive *S. stercoralis* infection in these patients. In a number of cases, both corticosteroid use and HTLV-1 status have been documented, during which severe *S. stercoralis* hyperinfection syndrome resulted from corticosteroid use in the presence of underlying HTLV-1, although rapid ivermectin therapy facilitated recovery [21,22]. It is therefore believed that combination of corticosteroids and HTLV-1 leads to more severe helminth infection, known as hyperinfection. However, corticosteroid use is by no means a requirement for co-infection. One male patient showed a unique manifestation of disease with infertility and scrotal pain [20]. In this specific case, *S. stercoralis* was detected in the ejaculate and HTLV-1 was positive, but no corticosteroid use was documented for this patient [20]. Similarly, a patient on chemotherapy for HTLV-1 associated leukemia, presented with *S. stercoralis*-related gastrointestinal (GI) symptoms, such as nausea and vomiting, and corticosteroid use was not documented.

A common theme of many of these case reports is that more severe symptoms of the helminth infection are often noted in the presence of HTLV-1 infection. Obstructive GI disease has been characterized in a number of other co-infected patients [16,17]. In one case, intestinal obstruction induced by *S. stercoralis* was observed in a pregnant woman following corticosteroid treatment, and it was subsequently determined that she was an HTLV-1 carrier [18]. Another report described a household in rural Brazil, in which the entire family was HTLV-1 positive, and the 13-year-old son exhibited eosinophilia and very large numbers of *S. stercoralis* worms, larvae, and eggs [19].

Although most individuals with HTLV-1 remain asymptomatic carriers, patients with the *S. stercoralis* co-infection are very often documented to have fully developed HTLV-1-associated diseases such as ATLL [23,24]. For example, one report described a patient with chronic strongyloidiasis who recently developed ATLL while on corticosteroid therapy and eventually developed disseminated *S. stercoralis* disease [25]. Other parasitic or viral infections often occur in these patients as well. One other report described a patient with scabies and ringworm infections, although it is not clear whether the dermal infections were the result of immune modulation by HTLV-1 [26]. Another patient had superimposed HIV-1 infection [27] while another had hepatitis B virus (HBV) [28]. HTLV-1 and HIV-1 have some similarities in their host target cells and modes of transmission, although HIV co-infection with *S. stercoralis* has not been as well-documented. Therefore HIV-1 patients with *S. stercoralis* should be screened for HTLV-1 as well. These scattered case reports, as well as some limited cohorts (see below) suggest the possibility of more severe disease presentations in co-infected patients, although definitive studies to prove this are yet to be done.

Some co-infected patients have presented with symptoms initially not suspected to be related to strongyloidiasis and without known HTLV-1 infection. For example, in one case, a patient with GI issues and suspected vasculitis was treated with steroids, and subsequently developed *S. stercoralis*, with poor response to ivermectin therapy, and ultimately was diagnosed with HTLV-1 infection [22]. Another patient with HBV and HTLV-1, who was undergoing corticosteroid and anti-viral therapy, showed a falsely negative *S. stercoralis* ELISA, presumably due to the profound immunosuppression [28]. He subsequently was diagnosed with *S. stercoralis* infection after receiving a hematopoietic stem cell transplant [28]. In another case, an HTLV-1 carrier presented with meningitis and liver abscess due to *Klebsiella*, along with chronic *S. stercoralis* infection. This combination of infections leads to a complicated and ultimately fatal clinical course [34]. These are some of the atypical presentations of the co-infection.

A number of case reports have raised the possibility that co-infection with HTLV-1 may alter the response to helminth treatment. Ivermectin is the standard treatment for *S. stercoralis* infection, while albendazole is sometimes included in combination as a second agent or used as monotherapy. Daily oral ivermectin is normally indicated, but subcutaneous ivermectin has shown success in patients with severe strongyloidiasis and HTLV-1 co-infection in whom oral medications were not tolerated or absorbed [31]. Interestingly, an HTLV-1 and *S. stercoralis* co-infected patient has been reported who failed to respond to ivermectin, requiring addition of albendazole to the therapeutic regimen; it is possible (see below) that the immunomodulatory effects of HTLV-1 may have contributed to the aggressive *S. stercoralis* infection [32]. Complications of disseminated disease associated with HTLV-1 and *S. stercoralis* may include *Klebsiella* meningitis, acute respiratory distress syndrome, and *Clostridium difficile* and *Escherichia coli* infections [21,32,33].

Case studies are useful for assessing clinical data but do not allow for robust mechanistic studies of disease pathogenesis. Regardless, several case studies, accompanied by careful laboratory analyses have provided insights into a potential mechanism for the effects of HTLV-1 and *S. stercoralis* co-infection. HTLV-1-associated leukemia patients show upregulation of CD25+/FOXP3+ T-cells which may increase risk for *S. stercoralis* co-infection [26]. IL-2 receptor expression has also been observed in these patients [26]. Recently, a patient with concurrent HTLV-1 and *S. stercoralis* infection was determined to have elevated IL-17 levels following ivermectin treatment [19]. More studies are needed to determine if IL-17 modulates the consequences of co-infection. Case studies provide a method to gauge the various presentations of the combined co-infection, provide insights for additional study, and provide illustrations of the rising incidence of HTLV-1 and the frequent co-infection with *S. stercoralis*.

## 4. The Impact of *S. stercoralis* on HTLV-1 Infection

Given early epidemiologic studies and the various cases reported in the literature, there appears to be a strong association of *S. stercoralis* infection with HTLV-1 infection [13,73]. Interactions between the pathogens may therefore change the course of each infection. Shortly after the discovery of HTLV-1 and *S. stercoralis* co-infection, Yamaguchi et al hypothesized that this interaction may be responsible for the earlier onset of ATLL seen in Caribbean and African patients (approximately 43 years [74]) as compared with ATLL onset in Japan (approximately 56 years [75]). Further data through the 1990s provided some support for the hypothesis that *S. stercoralis* co-infection is a co-factor for ATLL. Plumelle et al. reported a dramatic difference in age of onset of ATLL in *S. stercoralis* co-infected patients (median age 39 years) as compared with non-co-infected patients (median age 70 years, [76]). In this early study which was prior to more recent treatment modalities, the overall survival for co-infected individuals was independent of age and was more favorable for co-infected individuals (167 days) compared to HTLV-1 infection alone (30 days). There have been some interesting published findings that begin to explain possible mechanisms for *S. stercoralis* in altering the course of HTLV-1 infection. Specifically, *S. stercoralis* may have a role in changing both viral load and expression of immune-related genes, potentially worsening the clinical course of HTLV-1 infection.

The effect *S. stercoralis* has on HTLV-1 has often been assessed through comparison of HTLV-1 carriers with and without helminths. Gabet et al. used quantitative PCR methods to study HTLV-1 DNA proviral load in 18 patients with *S. stercoralis*+/HTLV-1+ along with 15 patients with HTLV-1 alone from French Guyana and Martinique [77]. Their main conclusion was that *S. stercoralis* infection stimulates the oligoclonal proliferation of HTLV-1-infected cells. This was hypothesized to account for the possibly shorter latency period in those that develop ATLL; expanding oligoclonal populations of HTLV-1 infected cells undergoing increased rounds of replication were hypothesized to have acquired additional cellular mutations enhancing the likelihood of malignant transformation. A mechanism of how *S. stercoralis* led to clonal expansion was not elucidated. As noted above, enhanced viral load has been associated with more severe infection and potential clinical manifestations. Gabet et al. looked only at HTLV-1 carriers without clinical manifestations of ATLL or HAM/TSP leaving open the possibility that the interactions between viral dynamics and nematode infection may differ in those already experiencing an HTLV-1-associated disease manifestation.

Subsequent studies have also correlated viral load and clonal proliferation of HTLV-1 in co-infected patients using a larger sample size, including individuals with HTLV-1-specific disease presentations. Applying the pioneering deep sequencing of HTLV-1 integrations sites, 61 individuals with HTLV-1 alone (i.e., no co-infection), were studied [78]. This included 14 asymptomatic HTLV-1 carriers, 26 HAM/TSP patients, 20 ATLL patients, and 1 with uveitis. In addition, 14 patients co-infected with *S. stercoralis* and HTLV-1 were included. This study had the advantage of samples representing of several clinical presentations of HTLV-1 patients, including uveitis and neurological diseases. An increased number of HTLV-1-infected clones was observed in *S. stercoralis* patients. In addition, there was higher proviral load in co-infected patients due to increased mean clone abundance (i.e., a greater abundance of individual HTLV-1-infected cell clones within the overall population of lymphocytes). This indicates that in the presence of *S. stercoralis* infection, either endogenous restrictions on clonal proliferation are over-ridden or there is some spur to increased proliferation in already proliferating clones. A surprising observation from this study was that the previously identified association between HTLV-1 infection and the genomic context of proviral integration, was not observed in proliferating clones in the setting of *S. stercoralis* co-infection. Both of these observations suggest that in the presence of *Strongyloides* co-infection there is an additional drive for proliferation of subsets of HTLV-1 infected cells, which in turn may provide a relevant contribution to the early observations of earlier onset of ATLL in co-infected patients.

In addition to the impact of *S. stercoralis* on HTLV-1 viral load, the immune response in these patients is of great interest. The shift to a Th1 response is well-documented in HTLV-1 carriers, and this is likely involved in the mechanism of co-infection with helminths because the body cannot induce an effective Th2 response against *S. stercoralis*. It is worth mentioning, however, that some studies have shown increased Th2 responses in HTLV-1 patients with the parasites compared to those without them. Porto et al. studied 310 HTLV-1 carriers and 32 HAM/TSP patients in Brazil [71]. Specifically, 35 HTLV-1 carriers co-infected with helminths were compared with a control group of 35 without helminths. Those co-infected had lower interferon gamma (IFN-γ), and increased IL-5 and IL-10; indicating an overall decreased Th1 response compared to HTLV-1 carriers, associated with increased Th2 response. This study also contrasts to other recent studies because the patients with the *S. stercoralis* showed a lower HTLV-1 proviral load. The lower viral burden may have been the reason for the predominant Th2 response rather than Th1. These observations raise questions as to whether *Strongyloides* always induces proliferation of HTLV-1-infected cells, and which T-cell response predominates.

Other work has sought to better understand the role of IL-2 in the mechanism of the co-infection. Satoh et al. studied 123 patients in Okinawa, showing that HTLV-1/*S. stercoralis* co-infected patients had higher proviral load and increased CD4+25+ T-cells, compared to patients with HTLV-1 alone [79]. This corroborates other work described above. Additionally, it was determined that *S. stercoralis* antigen activated the IL-2 promoter, supporting growth of cell lines with HTLV-1 or transduced with Tax. Overall, this was a new finding and showed that *S. stercoralis* can induce polyclonal expansion of HTLV-1 cells, through activation of the IL-2/IL-2R receptor system. Later work in Brazil came to similar findings through the study of 30 patients with *Strongyloides* and HTLV-1 and 60 patients with HTLV-1 only [80]. Co-infected patients showed significantly higher levels of secretory IL-2 receptor (sIL-2R) than patients with HTLV-1 alone, and also of interest, higher levels of sIL-2R were noted before treatment of *S. stercoralis*. Treatment of helminth infection decreased these levels, again suggesting a role of the parasites in modulating HTLV-1 infection. This further suggests a role of helminthic infection in enhancing T-cell proliferation through IL-2 induction and induction of IL-2 receptor levels when co-infected with HTLV-1. Further studies that might integrate this work on the effects of *S. stercoralis* on IL-2/IL-2R function with the apparent increased drive for HTLV-1 clonal abundance and proliferation in the setting of co-infection [81] will be of great interest and may help establish key mechanistic links between *S. stercoralis* infection and HTLV-1-associated disease. It also would have implications for both T-cell functions and T-cell oncogenesis. In addition to potential effects on oncogenesis, other studies are exploring the impact of HTLV-1 and *S. stercoralis* infections on the incidence of other malignancies. Co-infection has been independently associated with a number of different cancers other than ATLL; for example, HTLV-1 infection has been associated with increased non-ATLL lymphomas and hepatocellular carcinoma in a long-term follow-up of a cohort in Okinawa [82]. However, despite relatively common co-infection, it is unclear if co-infection increases the risk for non-ATLL cancers as well.

## 5. The Impact of HTLV-1 on the Immune Response to *S. stercoralis*

HTLV-1 appears to have specific effects on how the body reacts to *S. stercoralis* in patients with the co-infection. Clinically, this appears to be manifest as a significant association between HTLV-1 infection and the occurrence of the aggressive and potentially life-threatening clinical syndrome of *S. stercoralis* hyperinfection [81,83]. In particular, Gotuzzo et al. in a study from Lima, Peru demonstrated a startlingly high rate of HTLV-1 co-infection in patients with hyperinfection (almost 86%), but not in those with simple intestinal infestation, which did not differ in frequency between HTLV-1+ and HTLV-1 uninfected patients [83]. Similar results in a Brazilian population were reviewed by Carvalho [81]. The increased susceptibility of HTLV-1 and *S. stercoralis* co-infected patients to aggressive parasite infection, strongly suggests that HTLV-1 infection may compromise an effective host response limiting nematode infection. A series of studies have measured various cytokine and IgE levels to determine possible associations between cytokine production and parasite expulsion in co-infection. In typical immune responses to the presence of *S. stercoralis*, a Th2 response includes an increase in the production of IL-4, which promotes isotype switching in B cells and the production of IgE [84]. Subsequently, IgE primes eosinophils and increases the anti-parasitic response. In co-infection with HTLV-1, there is increased production of IFN-γ, resulting in a shift from a Th2 response to a Th1 response. Given that a Th2 response is necessary to expel the parasite, the presence of a Th1 response would exacerbate the phenotype of disease and symptoms related to the *S. stercoralis* infection.

**Figure 2:**
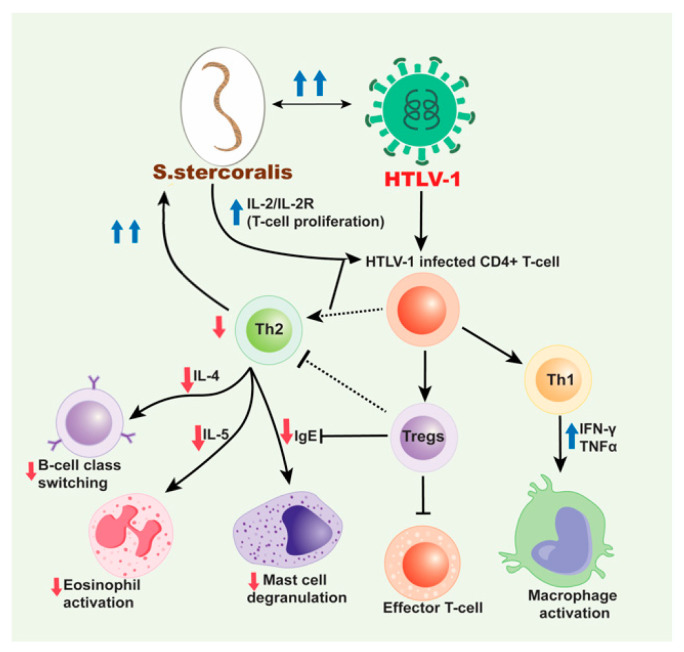
Mechanism for HTLV-1/*S. stercoralis* co-infection. HTLV-1 infected CD4+ T-cells induce a predominant Th1 response over a Th2 response and produce high production of interferon gamma (IFN-γ) and tumor necrosis factor alpha (TNF-α). This decreases the production of cytokines, IL-4 and IL-5 subsequently inhibiting B cell isotype switching to IgE and decreasing eosinophil activation, respectively and also decrease in the mast cell degranulation. The increased Th1 response also contributes to the decreased Th2 response with the release of IL-10. HTLV-1 has also been associated with an increase in Treg cells, which are CD4+CD25+FOXP3+ T-cells that lead to immunosuppression and downregulation. These cells inhibit effector T-cells and likewise help decrease the Th2 response seen in HTLV-1/*S. stercoralis* co-infection. With the decrease in IgE production and decreased eosinophil and mast cell activation and priming, the *S. stercoralis* parasite cannot be eliminated from the body. Therefore, with cases of co-infection, disseminated strongyloidiasis is likely. Moreover, the dissemination of *S. stercoralis* results in the IL-2/IL-2R which leads to increase in the polyclonal expansion of HTLV-1 infected T-cells which leads to these sequences of events.

Several studies observed that with increased production of IFN-γ characteristic of a Th1 response, HTLV-1 infection results in decreases in IL-4, IL-5, and IgE [84,85]. These changes in cytokine production, specifically the apparent decrease in IL-4 and IL-5, then decreases the immune response to the parasite. A 2002 study included 32 HTLV-1 positive and 47 HTLV-1 negative, *S. stercoralis*-infected individuals from Japan [79]. Further, 21 *S. stercoralis* negative individuals, of whom, 9 were HTLV-1 positive, were also included [86]. Patients were diagnosed with *S. stercoralis*, and the effect of treatment of strongyloidiasis in HTLV-1 carriers was observed. This study observed serum IgG4 levels were higher in patients with co-infection, supporting the hypothesis that IgG4 may block production of IgE. Increased IFN-γ and tumor growth factor beta (TGF-β) were also seen in co-infected patients, followed by a decrease in IL-4. As noted above IL-4 is required for B cells to undergo isotype switching to produce IgE. Another study examined the effect of HTLV-1 infection on serologic and skin tests for strongyloidiasis to assess the impact on IgE and eosinophils [85]. Data was collected from three groups, including HTLV-1 seropositive individuals identified at blood banks; residents of an *S. stercoralis* endemic area in Salvador, Brazil who tested positive for helminth infection via fecal examination; and patients hospitalized for severe infection. This study concluded that HTLV-1 reduced the sensitivity of parasite specific IgE testing as well as immediate hypersensitivity skin test. The decrease in IL-4 also apparently prevented an increase in intestinal fluid content associated with parasite rejection. These findings were in accordance with prior knowledge of the role of IL-4. Observed effects were attributed to the impaired isotype switching from IgG to IgE as illustrated in Figure 2. This overall shift away from a typical anti-parasitic response, results in less elimination of the *S. stercoralis* infection through eosinophil activation and specific immunoglobulin production. Subsequently, the patient may develop disseminated strongyloidiasis and further complications. The same data set was also used to measure various cytokine and IgE levels to determine other possible association between cytokine production and parasite expulsion. The results included a negative correlation between IFN-γ and total IgE, which was in accordance with predicted levels in co-infection. Other results included an increase in IL-10 and a decrease in IL-13, which corresponded with the expected Th2 to Th1 shift.

Despite many studies that showed a change in IgE production, a recent study found unchanged serum IgE levels with co-infection. The study observed Japanese patients with *S. stercoralis* treated between 1991–2014 [87]. Patients received treatment with ivermectin and despite HTLV-1 co-infection their serum IgE was unchanged. The two cohorts were used to collect data on symptoms and serum IgE and eosinophil counts. This study also demonstrated that serum IgE levels were lower than expected in female patients under 70 years old, indicating a potential relationship between sex and serum IgE levels. There was also a proposed impact of age on serum IgE levels, potentially contributing to the normal IgE levels seen in patients included in this study. Patients under 70 years of age typically showed normal IgE levels even with co-infection compared to individuals over 70 years of age. This particular study unfortunately does not appear to have measured IL-4/IL-5 levels to correlate to IgE. Additionally, few of these studies correlate the response to *Strongyloides* to HTLV-1 proviral load; therefore, it is unclear if worsening HTLV-1 infection causes an incremental impairment of immune response to the helminths.

The Th2 to Th1 shift is one of the potential mechanisms for the impact of HTlV-1 on the *S. stercoralis* infection. However, other changes in the CD4+ T-cell population have also been noted. A current area of interest is the impact HTLV-1 has on Treg cells. Several studies show associations between Treg cells and IgE expression, which may contribute to the pathogenic mechanisms of HTLV-1/*S. stercoralis* co-infection [88,89]. Montes et al. compared a cohort of co-infected patients, and in addition to observing higher parasite burden and higher HTLV-1 proviral loads, the relative proportion of Tregs was significantly higher than in patients infected with either agent alone [90]. HTLV-1 co-infection was associated with increased Tregs and decreased ex vivo *S. stercoralis* larval antigen-driven production of IL-5. This decrease in IL-5 production is potentially also followed by decreased eosinophil differentiation, decreased helminth killing, and decreased mast cell degranulation. Malpica et al. [91] extended these observations to determine how increased Tregs impacted the local immune response to *S. stercoralis* in the infected intestine. The study demonstrated increased overall CD3, CD8 and Treg cell responses in duodenal biopsies of co-infected patients versus non-specific duodenitis or normal patients. However, there was also a relative decrease of the aforementioned cells and eosinophils adjacent to the parasites. Recently, intriguing follow-up studies showed that successful treatment of *S. stercoralis* infection in co-infected patients resulted in decreased Tregs, however the defect in IL-5 production in response to *Strongyloides* antigen persisted [92]. Thus, HTLV-1 infection induces long-term and persistent deficits in the host response to *S. stercoralis* even following parasite eradication. Many of these findings corroborate the proposed role of HTLV-1 in this co-infection. The presence of the retrovirus results in increased Treg cells and a downregulation of effector T-cells, likely contributing to increased parasite burden (as depicted in Figure 2), although it is unclear if this is specific to the *S. stercoralis* species. However, future studies could help determine other immune responses to co-infection beyond cytokine production and Treg promotion as well as the potential confounding role of corticosteroid treatment in the impact of HTLV-1 on *S. stercoralis*.

## 6. Mouse Models for the Study of Immunology and Pathology of HTLV-1 and Strongyloidiasis

The lack of a suitable small animal model has hindered understanding of the immuno- and neuropathogenesis of HTLV-1 in an in vivo system, due to the inefficient fusion of HTLV-1 envelope with murine cells. This shortcoming was overcome by the development of an infectious chimeric virus [88,93], which was successfully utilized in our previous studies [89,94]. However, novel therapeutic strategies can be better tested in the context of humanized (hu) mice, which in recent years have played important roles in understanding of viral pathogenesis [95]. The improved BLT (bone marrow liver thymic) mice allow study of MHC-restricted T-cell responses against viral infections [96,97]. These mice show generation of B and T-cells, macrophages, NK cells and DCs along with appropriate human MHC restriction over at least 6 months homing to secondary lymphoid organs without being massively activated [98]. HTLV-1 infects CD34^+^ hematopoietic stem cells and can establish infection in NOD/SCID and NOD/SCID/γc^-/-^ (NSG) mice [99,100,101]. Chronic infection was demonstrated in cord blood-transplanted Rag/γc^-/-^ mice [102]. Fairly recently, we have also demonstrated HTLV-1 chronic infection in Rag1 and BLT mice in the context of HAM/TSP [103]. With respect to ATLL, HTLV-associated lymphomas have been recapitulated in CD34-reconstituted NSGkit mice [100,102]. For leukemia, a novel hu mouse model has been generated by the intra-bone marrow (IBM) injection of human CD133^+^ stem cells into NOD/Shi-scid/IL-2Rγc null (NOG) mice [104]. This model displays distinct ATL-like symptoms including hepatosplenomegaly, hypercytokinemia, oligoclonal proliferation of HTLV-1-infected T-cells and flower cells as well as specific antiviral immunity. IBMI-huNOG mice are well accepted as the current gold standard for evaluating anti-ATL drugs and vaccine candidates.

For *S. stercoralis*, various mechanisms of innate and adaptive protective immunity have been studied in mouse models. In naïve mice, parasite-released soluble factors recruit neutrophils, eosinophils, and macrophages directly to the parasite. Neutrophils and eosinophils are then able to kill L3 through the release of myeloperoxidase and major basic protein, respectively. However, both of these granulocytes require complement factor C3b to kill the larvae. After the neutrophils die, they also release extracellular traps (NETs) consisting of a fibrous network of nuclear DNA. The worms are trapped within the NETs, preventing their movement and facilitating the attachment of slow-moving effector cells to the rapidly migrating worms [105,106]. After killing and disrupting the worms, eosinophils also act as antigen presenting cells, processing the parasite antigens and inducing a Th2 response. Adaptive immunity to the larval stages of *S. stercoralis* is highly efficient and is dependent on CD4+ Th2 cells for the release of IL-4 and IL-5. B cells, in particular B-1 cells, are also required for adaptive immunity, resulting in the production of immunoglobulins necessary for neutrophil and macrophage mediated killing. Whereas neutrophils require complement and IgG to kill the larvae through antibody-dependent cellular cytotoxicity (ADCC), macrophages require IgM to kill the larvae independent of ADCC. While eosinophils do not act as effector cells in this response, their role as antigen presenting cells is vital to the production of antigen-specific IgM. Finally, mice infected with *S. stercoralis* developed alternatively activated macrophages within the peritoneal cavity after exposure to the cytokine IL-4. Alternatively, activated macrophages, but not classically activated macrophages, kill the larvae in collaboration with neutrophils and complement. Thus, the adaptive immune response kills through two antibody dependent mechanisms, IgG with neutrophils and IgM with macrophages, and one antibody independent mechanism consisting of macrophages, neutrophils, and complement [105,106].

It is clear from these studies that the innate and adaptive immune responses in mice are highly efficient at controlling the infection. It was hypothesized that in the absence of a functional immune response, *S. stercoralis* infection would develop in mice. To test this hypothesis, severe combined immunodeficiency (SCID) mice, which lack T and B cells, were first used to establish that mice are susceptible to infection with *S. stercoralis*. L3 of *S. stercoralis* were injected into SCID mice, yielding a small number of adult worms [107]. To further test the hypothesis that the immune response controls susceptibility of mice to infection with *S. stercoralis*, L3 were injected into NSG mice, which have a profound deficiency in both innate and adaptive immunity. It was postulated that in the absence of a functional immune response, infection and hyperinfection with *S. stercoralis* would develop in NSG mice. While large numbers of adult worms and L1 consistently developed in the NSG mice, no signs of hyperinfection were detected. Treatment of NSG mice to eliminate residual neutrophils likewise did not result in hyperinfection, suggesting that the residual innate immune response was not responsible for controlling its development. Infected NSG mice were then treated with methylprednisolone acetate, a drug associated with the development of hyperinfection in humans, to determine if the drug would function in a similar manner in the infected NSG mice. Subsequently, *S. stercoralis*-infected NSG mice treated with methylprednisolone acetate displayed increased mortality rates, a greater than 10-fold increase in adults and L1, and the development of large numbers of L3a, consistent with a hyperinfection-like phenotype. Several hypotheses were generated to explain how hyperinfection developed in NSG mice in the presence of methylprednisolone acetate. One possibility is that the methylprednisolone acetate induced this phenomenon by eliminating a residual element of the immune response retained by the NSG mice. An alternative explanation was that the methylprednisolone acetate had a direct effect on the worms driving their development into L3a and thus the development of hyperinfection. This second explanation was further investigated by treating the NSG mice with methylprednisolone acetate-induced hyperinfection with an agonist to the parasite nuclear receptor *Ss*-DAF-12. Following this treatment, these NSG mice had significantly reduced worm burdens, opening the door to novel new treatments for this potentially fatal infection in humans [108]. Methylprednisolone acetate induces hyperinfection in gerbils infected with *S. stercoralis* [109]. However, the relationship between HTLV-1 and *S. stercoralis* cannot be studied in the Jird model. Since humanized immunodeficient mice are susceptible to infection with HTLV-1 [104,110,111] and these mice are also susceptible to infection with *S. stercoralis,* it may be possible to ask novel questions regarding the relationship between these two infections agents and the development of hyperinfection. Further studies should examine if *S. stercoralis* hyperinfection develops in NSG mice co-infected with HTLV-1. If it is possible to simulate this co-infection, the *S. stercoralis* infection could be further characterized to observe if L3a, with their characteristic size and morphology, develop in this model. Other directions include identifying shared and unique features in the hyperinfections induced by methylprednisolone acetate and HTLV-1. With these results, further research could determine if an antagonist to the parasite nuclear receptor *Ss*-DAF-12 may be effective at blocking hyperinfection induced by HTLV-1 in mice co-infected with *S. stercoralis* and HTLV-1.

## 7. Conclusions and Future Perspectives

HTLV-1 exhibits a wide variety of disease manifestations, although most infected individuals remain asymptomatic carriers. Even in carriers, the virus is a risk factor for the development of the disseminated hyperinfection of *S. stercoralis*. The co-infection is well documented in several infected populations and has been characterized both through a number of case reports, a handful of cohort studies, and some mechanistic studies. Similar to the individual infections, the co-infection affects a variety of patient demographics and ages. It is also common for the co-infection to manifest many years after exposure to HTLV-1. Mechanistically, it is proposed that HTLV-1 alters the immune response to *S. stercoralis* by causing a Th2 to Th1 shift. This causes diminished IgE and decreased IL-4 and IL-5, resulting in difficulty mounting an effective anti-helminth response and predisposing to more severe infection. It is possible that IgG4 blocks IgE production, while IFN gamma decreases IL-4 and IgE. Treg cells may also have a role lowering eosinophil counts. When comparing co-infected patients to those with HTLV-1 alone, *S. stercoralis* appears to increase the proviral load of HTLV-1 through induction of clonal proliferation. Epidemiologic studies have suggested a potentially early onset of HTLV-1 induced ATLL. An intriguing possibility, needing considerably more study is that this may be mediated through activation of IL-2 promoter in infected cells, potentially enhancing clonal proliferation and also resulting increased amounts of soluble IL-2R. This may in turn have numerous effects on immune homeostasis. Case studies show that carriers of HTLV-1 with strongyloidiasis often present with additional infections, causing meningitis, gastrointestinal symptoms, or even respiratory distress. Furthermore, severe disseminated strongyloidiasis, which can infect systems other than the gastrointestinal system, and hyperinfection syndrome, which is often seen in HTLV-1 patients who receive corticosteroids [43] are more prevalent in the setting of HTLV-1 co-infection. Given the possibility for such severe infections, it is clinically imperative to screen for *S. stercoralis* infection in HTLV-1 carriers through PCR detection using stool samples [112]. Upon identification of these cases, anti-parasite medication ivermectin is still the first-line treatment for *S. stercoralis*. However, sometimes ivermectin is given for a longer course, or even combined with albendazole [32]. Additional treatments are being explored such as drugs targeting the Ss-DAF-12 receptor in mouse models.

There are a number of important areas for future study of HTLV-1 and *S. stercoralis* co-infection. The effects of *S. stercoralis* and HTLV-1 co-infection have been largely characterized in HTLV-1 carriers; relatively few studies have looked at presentations and outcomes of *S. stercoralis* in ATLL and HAM/TSP patients. One patient with ATLL and *S. stercoralis* experienced a severe response sepsis and organ failure following manifestation of symptoms [24]. As previously described, an earlier study examined 38 ATLL patients in Martinique, 19 with the *S. stercoralis* co-infection, and the rest having ATLL alone [76] and showed that the co-infected patients survived longer than ATLL patients without *S. stercoralis*. Given the short study and its performance prior to many of the newer therapeutic strategies for treating ATLL, it is difficult to determine the clinical significance of this difference in the current treatment setting. Subsequent work by this group focused on 11 ATLL patients with four being diagnosed with *S. stercoralis* and seven without the co-infection [113]. Co-infection in ATLL patients led to higher response rate to chemotherapy, with all 4 co-infected patients having complete remission and an overall longer survival [113]. Although this is a small sample, it does raise interesting questions about how ATLL prognosis may be positively affected by co-infection. Another area of surprising discrepancy is that some studies have shown lower proviral load in HTLV-1 patients with *S. stercoralis*, particularly in the context of HAM/TSP. One study in particular observed low proviral load in a few patients manifesting HAM/TSP and proposed that helminths could inhibit HTLV-1 transcription and prevent progression to HAM/TSP [71]. They also proposed the suppression of the HTLV-1 Th1 response may contribute to the decreased proviral load in these patients. Few patients with HAM/TSP have been reported with co-infection, resulting in limited data to assess the precise impact of *S. stercoralis*.

Another area of research priority is the development of robust models that will allow study of the details of pathogenic interactions between HTLV-1 and *S. stercoralis*. As described above, mouse models are limited both in their ability to mimic the long-term pathogenesis of HTLV-1 infection and to probe the details of the host immune reaction to *S. stercoralis*. A particular problem is that the models of HTLV-1 infection in different mice carrying human CD4+ T-cells are often associated with a rapid progression from infection to a full-blown leukemic phenotype. This limits the ability to study the evolution of *S. stercoralis* infection due to deficits in the reconstituted human immune system before the onset of the florid HTLV-1 proliferative disease. Rabbits have been successfully infected with HTLV-1 and have been a long-standing model of in vivo viral replication (reviewed in [114,115]); however full-fledged HTLV-1 diseases are not reproducibly observed in infected rabbits, limiting their utility. An intriguing, but expensive and relatively unavailable model is the simian T-cell leukemia virus type 1 (STLV-1), variants of which infect a wide variety old world monkeys and apes (reviewed in [116]). Chronic infection, often asymptomatic and only progressing to an ATLL-like phenotype in a subset of animals, is a hallmark of STLV-1 infection. In that respect, this models human infections with HTLV-1 quite well. Furthermore, infection of certain monkey strains such as the squirrel monkey, *Siamiri sciureus*, can lead to a chronic ATLL-like phenotype in two inoculated monkeys [117]. Whether these monkey models may play a useful role in probing HTLV-1 (or STLV-1) and *S. stercoralis* pathogenic interactions will only be determined by experimental modeling of co-infections in animals susceptible to both pathogens. A potential model for such a co-infection system could be marmoset species as recent studies have shown that they experimentally infectable by both HTLV-1 [118] and *S. stercoralis* [119].

Finally, as better animal models develop, it will be essential to examine potential pathogenic interactions of HTLV-1 and *S. stercoralis* in the intestine, as this is the anatomic locus most likely to be the focus of these interactions. As the site of *S. stercoralis* invasion that is the hallmark of the hyperinfection syndrome associated with HTLV-1 co-infection and other immunosuppressed states, the intestine is likely the key element in regulating a transition to the hyperinfection state. As noted above, there is limited but very intriguing recent preliminary data from co-infected patients [91] showing that co-infected patients exhibited increased numbers of CD3, CD8, and Treg cells in duodenal biopsies as compared with patients with non-specific duodenitis or normal patients; however, the numbers of these cells adjacent to the parasites was reduced. Overall, the proportion of Tregs was increased. It is difficult to evaluate the role of HTLV-1 infection in these changes in this preliminary study, as there were no direct comparisons to HTLV-negative *Strongyloides*-infected patients, or conversely duodenal biopsies from HTLV-1 infected patients. In fact, there is a striking lack of information about HTLV-1 infection of gut-associated lymphoid tissue. This is quite surprising considering that infection of the gut is the major route of HTLV-1 infection (through breast milk transmission). Furthermore, for HIV, the gut-associated lymphoid tissue with its high levels of CD4 T-cells is the major site of early viral replication [120]. In fact, while the ability of HTLV-1 to cross an epithelial barrier by transcytosis has been shown [121], there has been no direct demonstration of HTLV-1 infection of gut-associated lymphoid tissue. In this regard, the rabbit model of HTLV-1 infection, shown to exhibit many similarities of GALT with humans [122], may offer a tractable model for exploring HTLV-1 and *S. stercoralis* co-infection of the intestine.

As the prevalence of HTLV-1 increases in certain parts of the world [123,124], it appears likely that HTLV-1 and *S. stercoralis* co-infection will also increase providing both a new urgency as well as increased opportunities to study the pathogenic effects of co-infection. Overall, it is clear that HTLV-1-infected individuals are at an increased risk of developing severe disseminated strongyloidiasis. Therefore, further attention must be given to screening and treating these patients. Conversely, although data are less robust, there are both clinical studies as well as proposed mechanisms that would support an earlier onset of ATLL in co-infected patients. In order to develop new treatments that may lessen the disease burden for this growing patient population, further research is needed to truly determine the intricate role these microorganisms play as partners in the pathogenesis of HTLV-1 and *S. stercoralis* co-infection.

## Abbreviations

HTLV-1	Human T-cell leukemia/lymphoma virus type 1
HIV-1	Human immunodeficiency virus type 1
ATLL	Adult T-cell leukemia/lymphoma
HAM	HTLV-associated myelopathy
TSP	Tropical spastic paraparesis
STH	Soil transmitted helminth
L1	First stage larvae
L3	Third stage larvae
L3a	Autoinfective third stage larvae
IL-2	Interleukin-2
sIL-2R	Secretory interleukin-2 receptor
IFN-γ	Interferon gamma
IgE	Immunoglobulin E
TGF-β	Tumor growth factor beta
CCR4	C-C chemokine receptor type 4
Th2	Type 2 helper T-cell
NET	Neutrophil extracellular trap
ADCC	Antibody-dependent cellular cytotoxicity
SCID	Severe combined immunodeficiency
HBV	Hepatitis B virus
